# Nonsecretor Histo–blood Group Antigen Phenotype Is Associated With Reduced Risk of Clinical Rotavirus Vaccine Failure in Malawian Infants

**DOI:** 10.1093/cid/ciy1067

**Published:** 2018-12-18

**Authors:** Louisa Pollock, Aisleen Bennett, Khuzwayo C Jere, Queen Dube, Jonathan Mandolo, Naor Bar-Zeev, Robert S Heyderman, Nigel A Cunliffe, Miren Iturriza-Gomara

**Affiliations:** 1 Centre for Global Vaccine Research, Institute of Infection and Global Health, University of Liverpool, United Kingdom; 2 Malawi Liverpool Wellcome Trust Clinical Research Programme, University of Malawi, Blantyre; 3 Medical Laboratory Sciences Department, University of Malawi, Blantyre; 4 Department of Paediatrics, College of Medicine, University of Malawi, Blantyre; 5 International Vaccine Access Center, Department of International Health, Bloomberg School of Public Health, Johns Hopkins University, Baltimore, Maryland; 6 Division of Infection and Immunity, University College London, United Kingdom; 7 National Institute for Health Research Health Protection Research Unit in Gastrointestinal Infections, University of Liverpool, United Kingdom

**Keywords:** rotavirus, HBGA, vaccine, immunogenicity, Malawi

## Abstract

**Background:**

Histo–blood group antigen (HBGA) Lewis/secretor phenotypes predict genotype-specific susceptibility to rotavirus gastroenteritis (RVGE). We tested the hypothesis that nonsecretor/Lewis-negative phenotype leads to reduced vaccine take and lower clinical protection following vaccination with G1P[8] rotavirus vaccine (RV1) in Malawian infants

**Methods:**

A cohort study recruited infants receiving RV1 at age 6 and 10 weeks. HBGA phenotype was determined by salivary enzyme-linked immunosorbent assay (ELISA). RV1 vaccine virus shedding was detected by quantitative real-time polymerase chain reaction (qRT-PCR) in stool collected on alternate days for 10 days post-immunization. Plasma rotavirus–specific immunoglobulin A was determined by ELISA pre- and post-immunization. In a case-control study, HBGA phenotype distribution was compared between RV1-vaccinated infants with RVGE and 1:1 age-matched community controls. Rotavirus genotype was determined by RT-PCR.

**Results:**

In 202 cohort participants, neither overall vaccine virus fecal shedding nor seroconversion differed by HBGA phenotype. In 238 case-control infants, nonsecretor phenotype was less common in infants with clinical vaccine failure (odds ratio [OR], 0.39; 95% confidence interval [CI], 0.20–0.75). Nonsecretor phenotype was less common in infants with P[8] RVGE (OR, 0.12; 95% CI, 0.03–0.50) and P[4] RVGE (OR, 0.17; 95% CI, 0.04–0.75). Lewis-negative phenotype was more common in infants with P[6] RVGE (OR, 3.2; 95% CI, 1.4–7.2).

**Conclusions:**

Nonsecretor phenotype was associated with reduced risk of rotavirus vaccine failure. There was no significant association between HBGA phenotype and vaccine take. These data refute the hypothesis that high prevalence of nonsecretor/Lewis-negative phenotypes contributes to lower rotavirus vaccine effectiveness in Malawi.

## Teaser

In case-control and cohort studies in Malawi, we found little evidence that nonsecretor/Lewis-negative histo–blood group antigen phenotypes are associated with reduced rotavirus vaccine take in Malawian infants. Nonsecretor phenotype was associated with reduced risk of clinical vaccine failure.

Introduction of rotavirus vaccines into childhood immunization programs has reduced global child deaths from diarrheal disease [1], but current vaccines are less effective in low-income, high-mortality countries than in higher-income settings [2]. Multiple explanations for this disparity have been proposed, but definitive data are lacking [3]. A widely proposed hypothesis is that histo–blood group antigen (HBGA) phenotype could affect the replication of live rotavirus vaccines in the gut, potentially explaining observed population differences in rotavirus vaccine immunogenicity and effectiveness [4–9].

HBGA are complex carbohydrates expressed on the surface of red blood cells and mucosal epithelial cells. Secretion of HBGA, as free oligosaccharides in saliva and other exocrine secretions, is determined by expression of the *FUT2* gene. Mutations of *FUT2* result in a nonfunctional enzyme and “nonsecretor” phenotype. A combination of *FUT2* and *FUT3* gene expression determines the Lewis HBGA phenotype [10].

Rotavirus is a double-stranded RNA virus comprising an 11-segment genome in a triple-layer protein capsid. Rotaviruses are classified by capsid protein G (glycoprotein VP7) and P (protease-sensitive VP4) genotypes. HBGA glycans have been shown to bind in a strain-specific pattern to the VP8* subunit of VP4 [11–15]. In addition, epidemiological studies have shown that HBGA phenotype determines strain-specific susceptibility to rotavirus gastroenteritis (RVGE). Secretor and Lewis-positive phenotypes have been associated with increased risk of P[8] and P[4] RVGE [5, 7, 13, 16–20] and Lewis-negative phenotype with increased risk of P[6] RVGE [5, 7].

Both the monovalent human rotavirus vaccine Rotarix (RV1) and pentavalent human-bovine reassortant vaccine Rotateq are based on attenuated P[8] strains. HBGA-associated resistance to P[8] vaccine virus replication could therefore diminish vaccine response. Evidence to support this hypothesis is limited and inconsistent, and no data are available from sub-Saharan Africa [5, 6, 8]. Malawi is a low-income country that introduced RV1 nationally in 2012. Malawi has high rotavirus genotypic diversity, with around 20% of RVGE caused by P[6] strains [21]. Rotavirus vaccine effectiveness in the first year of life is estimated at 70% [22]. In this population, we sought to test the hypothesis that intrinsic resistance of Lewis-negative/nonsecretors to G1P[8] infection results in reduced immunoglobulin A (IgA) response, reduced vaccine virus replication, and impaired clinical protection against severe RVGE following G1P[8] rotavirus vaccine.

## METHODS

The relationships between HBGA phenotype, vaccine virus replication, and rotavirus-specific IgA response were determined in a longitudinal cohort study. The relationship between HBGA phenotype and clinical rotavirus vaccine failure was determined by a cross-sectional case-control study. The University of Malawi College of Medicine (P.09/14/1624) and University of Liverpool (00758) research ethics committees provided ethical approval for both studies.

### Study Population

#### Longitudinal Cohort Study

Healthy infants attending a vaccination clinic in Blantyre, Malawi, were consecutively recruited from April 2015 to August 2016, prior to first RV1 immunization, following informed parental consent. Blood samples were taken prior to the first RV1 dose (at approximately 6 weeks of age) and 2 weeks following the second RV1 dose (at approximately 12 weeks of age). Stool samples were taken on days 4, 6, 8, and 10 post-immunization.

#### Case-control Study

Infants aged between 10 weeks and 1 year with severe gastroenteritis, defined as Vesikari score ≥11 [23], were consecutively recruited from January 2015 to January 2017, with informed parental consent, from a secondary referral hospital and 3 primary healthcare centers in Blantyre, Malawi. Stools were tested for rotavirus by rapid immunochromatography test (RotaStrip, Coris Bioconcept, Belgium). Infants who tested rotavirus positive were recruited as RVGE cases (vaccine failures). Age-matched community controls without diarrhea (for at least 1 week prior to recruitment), born within ±30 days of RVGE cases, were recruited from randomly generated locations within the healthcare catchment areas of each recruitment site in a 1:1 ratio. All cases and controls had received 2 doses of RV1 vaccine, confirmed by hand-held health records.

### Data Collection and Anthropometry

Socioeconomic and demographic data were collected by structured interview. Nutritional status was determined by measurement of length, weight, and mid-upper arm circumference (MUAC; a measure of wasting) at time of recruitment compared to World Health Organization age-determined *z* scores [24].

### Laboratory Methods

For detailed laboratory methods see Supplementary Methods. HBGA phenotyping was determined by detection of antigens A, B, H, and Lewis a and b in saliva by enzyme-linked immunosorbent assay (ELISA), using specific monoclonal antibodies, detected by peroxidase conjugated anti-mouse IgM. Infants with detectable salivary A, B, or H antigen were classified as secretors. Where detection of A, B, and H antigens was negative or borderline, secretor status was confirmed by ELISA to detect lectin antigen [25]. Infants who were positive for either Lewis a or Lewis b antigen were classed as Lewis positive, and those negative for both Lewis antigens as Lewis negative. *FUT2* genotype was determined for infants of nonsecretor phenotype with enough blood available. DNA was extracted from whole blood using the Qiagen DNA Blood Mini Kit (Qiagen, Germany) in accordance with manufacturer’s instructions. *FUT2* was amplified by polymerase chain reaction (PCR) and restriction fragment length polymorphism was used to identify inactivating mutations.

RV-specific IgA was determined by a custom antibody-sandwich ELISA [26]. Quantification was made by comparison to a standard plasma [27], reported as geometric mean concentration (GMC) in units per milliliter.

Nucleic acid was extracted from stool using the Qiagen Viral RNA Mini-Kit (Qiagen, Germany). Reverse transcription using random primers was used to generate complementary DNA [28]. RV1 shedding was determined by vaccine-specific non-structural protein 2 (NSP2) real-time PCR (RT-PCR) [29] and confirmed by VP6 quantitative RT-PCR (qRT-PCR) [30] (S1), with a cycle threshold (Ct) cutoff value for positivity of <40 cycles. In case-control study participants, including community controls, rotavirus infection was defined as VP6 ≥100 copies/mL by qRT-PCR. In both cases and in asymptomatic rotavirus infections in controls, rotavirus genotyping was undertaken using 2-stage RT-PCR [31].

### Statistical Analyses

All statistical analyses were performed in StataIC version 13.1 (StataCorp, College Station, TX).

#### Cohort Study

RV1 vaccine virus shedding was defined as 2 or more NSP2-positive, VP6-positive samples post-immunization. NSP2-positive, VP6-negative samples were considered negative. NSP2-negative, VP6-positive samples were assumed to reflect wild-type infection. A minimum of 2 post-immunization samples were required for inclusion in shedding analysis. Seropositivity was defined as RV-specific IgA >20 U/mL. Seroconversion was defined as a change from seronegative pre-immunization to seropositive post-immunization or at least a 4-fold rise in RV-specific IgA concentration post-immunization among infants seropositive at baseline. The relationships between HBGA phenotype (defined categorically on secretor and Lewis status) and these categorical outcomes were assessed by log-binomial regression. The relationships between HBGA phenotype and continuous variables (peak vaccine virus shedding, RV-specific IgA GMC) were determined by Wilcoxon rank sum test.

For the cohort study, a sample size of 200 was estimated to achieve 80% power to detect a risk ratio of 0.5 (vs equal risk, alpha 0.05).

#### Case-control Study

The odds of specific HBGA phenotype (defined categorically on secretor and Lewis status) were compared between cases and matched community controls by conditional logistic regression. With 1:1 controls, a sample size of 123 cases was estimated to achieve 80% power to detect an odds ratio (OR) of 2.5 (vs equal odds, alpha 0.05).

#### Genotyping Analysis

In an additional case-control analysis, the distribution of HBGA phenotype by genotype-specific RVGE was compared to community controls. This stratified analysis was unmatched, as there were too few matched pairs for meaningful analysis. Separate analyses determined distribution of HBGA phenotype in P[8], P[4], and P[6] RVGE compared to community controls by logistic regression. Rotavirus cases where genotype could not be confirmed were excluded.

A descriptive analysis of HBGA phenotype distribution in genotype-specific asymptomatic rotavirus infection in community controls was made.

## RESULTS

### Cohort Study

#### HBGA Phenotype, RV1 Fecal Shedding, and Seroconversion

A total of 293 infants were recruited to the cohort study. Of these, 243 infants in the first dose period, 214 infants in the second dose period, and 202 infants in both dose periods provided at least 2 stool samples. Both pre- and post-immunization samples for RV-specific IgA were provided by 196 infants. Demographic characteristics were similar in those with complete data compared to those with incomplete data (Supplementary Tables 1 and 2).

Compared to secretor infants, nonsecretors had significantly reduced risk of vaccine virus fecal shedding in the first dose period, but not in the second. The overall risk of vaccine virus fecal shedding in infants with data for both dose periods did not differ between nonsecretors and secretors (Table 1).

**Table 1.** Vaccine Virus Shedding and Rotavirus-specific Immunoglobulin A Response by Secretor Phenotype

Measure of Rotavirus Vaccine Response	Secretor	Nonsecretor	Risk Ratio^a^ (95%CI)	*P* Value
Vaccine virus shedding first dose period n, % (95% CI)	63/188, 34 (27%–41%)	10/55, 18 (10%–31%)	0.54 (0.3–0.98)	.04
Vaccine virus shedding second dose period n, % (95% CI)	58/169, 34 (27%–42%)	12/45, 27 (15%–42%)	0.78 (0.5–1.3)	.35
Overall vaccine virus shedding n, % (95% CI)	86/157, 55 (47%–62%)	18/45, 40 (26%–55%)	0.73 (0.5–1.1)	.11^b^
Peak vaccine virus shedding^c^ first dose period median Ct (IQR)	29.3 (25.9–32.3)	31.9 (30.4–34.1)	…	.13^d^
Peak vaccine virus shedding^c^ second dose period median Ct (IQR)	32.4 (30.6–34.7)	34.1 (31.9–35.0)	…	.21^d^
Seroconversion n, % (95% CI)	41/151, 27 (21%–35%)	6/45, 13 (6%–27%)	0.50 (0.2–1.1)	.08^b^
Post-immunization rotavirus-specific immunoglobulin A^e^ geometric mean concentration (95% CI)	109.3 (78.7–151.8)	81.3 (47.9–137.9)	…	.52^d^

Abbreviations: CI, confidence interval; Ct, cycle threshold; IQR, interquartile range.

^a^Risk ratio of vaccine virus fecal shedding/seroconversion in nonsecretor infants compared to secretor infants.

^b^Log-binomial regression.

^c^Peak vaccine virus shedding based on minimum non-structural protein 2 (NSP2) real-time polymerase chain reaction Ct value detected within dose period.

^d^Wilcoxon rank sum test.

^e^Only infants with detectable post-immunization rotavirus-specific immunoglobulin A >20 U/mL were included for analysis. This included 24/151(30%, 95% CI, 23%–38%) secretor and 9/45 (20%, 95% CI, 10%–35%) nonsecretor infants.

In a stratified analysis comparing shedding by sampling day, nonsecretors had significantly reduced risk of vaccine virus shedding (4/49, 8%) compared to secretors (51/182, 28%) on day 10 following the first vaccine dose. Risk of vaccine virus shedding was not significantly different between nonsecretors and secretors on other sampling days in the first dose period or on any day in the second dose period (Supplementary Table 3). There was no difference in peak level of vaccine virus shedding, as determined by NSP2 Ct value, by secretor status (Table 1). When Ct values were compared by sample day, median Ct values in nonsecretors were higher (viral load lower) compared to secretors on days 6 and 8 following the first vaccine dose, but not on any other sample day (Supplementary Table 4).

There was no difference in vaccine virus fecal shedding between Lewis-negative and Lewis-positive infants by any categorical or quantitative measure (Table 2, Supplementary Tables 3 and 4).

**Table 2.** Vaccine Virus Shedding and Rotavirus-specific Immunoglobulin A Response by Lewis Phenotype

Measure of Rotavirus Vaccine Response	Lewis Positive	Lewis Negative	RR^a^ (95%CI)	*P* Value
Vaccine virus shedding first dose period n, % (95% CI)	59/193, 31 (24%–37%)	14/50, 28 (17%–42%)	0.92 (0.56–1.5)	.73
Vaccine virus shedding second dose period n, % (95% CI)	57/169, 34 (27%–41%)	13/45, 29 (17%–44%)	0.86 (0.52–1.4)	.55
Overall vaccine virus shedding n, %, RR (95% CI)	84/159, 53 (45%–61%)	20/43, 47 (32%–62%)	0.88 (0.6–1.3)	.48^b^
Peak vaccine virus shedding^c^ first dose period median Ct (IQR)	29.8 (26.4–32.4)	31.2 (28.0–34.0)	…	.41^d^
Peak vaccine virus shedding^c^ second dose period median Ct (IQR)	32.1 (30.6–34.7)	33.9 (32.7–35.4)	…	.15^d^
Seroconversion n, %, RR (95% CI)	35/149, 24 (17%–31%)	12/47, 26 (15%–40%)	1.1 (0.6–1.9)	.77^b^
Post-immunization rotavirus-specific immunoglobulin A^e^ geometric mean concentration (95% CI)	114.5 (84.7–154.9)	74.5 (35.2–157.6)	…	.17^d^

Abbreviations: CI, confidence interval; Ct, cycle threshold; IQR, interquartile range; RR, risk ratio.

^a^Risk ratio of vaccine virus fecal shedding/seroconversion in Lewis-negative infants compared to Lewis-positive infants.

^b^Log-binomial regression.

^c^Peak vaccine virus shedding based on minimum non-structural protein 2 (NSP2) real-time polymerase chain reaction Ct value detected within dose period.

^d^Wilcoxon rank sum test.

^e^Only infants with detectable post-immunization rotavirus-specific immunoglobulin A >20 U/mL were included for analysis. This included 42/149 (28%, 95% CI, 21%–36%) Lewis-positive and 12/47(26%, 95% CI, 15%–40%) Lewis-negative infants.

Paired serological data were available for 196 cohort infants. Of these infants, 47 (24%) seroconverted. Eleven (6%) infants were seropositive at baseline. The risk of seroconversion was similar in baseline seropositive infants compared to baseline seronegative infants (risk ratio, 0.75; 95% confidence interval [CI], 0.21–2.7; *P* = .66). The risk of seroconversion did not differ by secretor or Lewis phenotype (Tables 1 and 2).

Among infants with detectable post-immunization RV-specific IgA, there was no difference in GMC between secretors and nonsecretors or between Lewis-positive and Lewis-negative infants (Tables 1 and 2).

In a sensitivity analysis where secretor/nonsecretor status was recategorized by confirmatory *FUT2* genotyping and phenotype at age 10 weeks, there remained no association between nonsecretor status and either vaccine virus shedding or seroconversion (Supplementary Table 5). Concordance between genotype and phenotype was 90%.

There was no difference in vaccine virus shedding or seroconversion when secretor phenotype was stratified by Lewis phenotype (Supplementary Table 6). In a subanalysis of secretor infants, there was no association between ABO phenotype and either vaccine virus shedding or seroconversion (Supplementary Tables 7 and 8).

### Case-control Study

A total of 119 eligible severe RVGE cases and 119 age-matched community controls were recruited. Median MUAC was lower in RVGE cases (13.1 cm; interquartile range [IQR], 12.4–14 cm) than in community controls (13.8 cm; IQR, 13.2–14.5 cm; *P* < .01). No other differences in anthropometric or socioeconomic characteristics between cases and controls were observed (Supplementary Table 9).

#### HBGA Phenotype Distribution in Infants With RV1 Clinical Vaccine Failure

The prevalence of nonsecretor phenotype was significantly lower in infants with clinical RV1 vaccine failure (14/119, 12%) compared to community controls (33/119, 28%). The odds of nonsecretor phenotype were more than 60% lower in RV1 vaccine failures than in age-matched community controls (Table 3). In a sensitivity analysis where secretor/nonsecretor status was recategorized by *FUT2* genotyping, the distribution of nonsecretor phenotype in RV1 vaccine failures and controls was unchanged (OR, 0.36; 95% CI, 0.17–0.74; Supplementary Table 10). Concordance between genotype and phenotype was 86%.

**Table 3.** Histo–blood Group Antigen Phenotype Distribution in Rotavirus Vaccine Failures and Community Controls

Histo–blood Group Antigen Phenotype	Prevalence in Rotavirus Gastroenteritis Cases (Vaccine Failures) n, % (95% CI)	Prevalence in Community Controls n, % (95% CI)	Odds Ratio^a^ (95% CI) *P* Value
Nonsecretor	14/119, 12 (7%–19%)	33/119, 28 (20%–37%)	0.39 (0.20–0.75) *P* = .005
Lewis negative	24/119, 20 (14%–28%)	31/119, 26 (19%–35%)	0.70 (0.37–1.3) *P* = .27

Abbreviation: CI, confidence interval.

^a^Odds ratio of nonsecretor/Lewis-negative phenotype in vaccine failures compared to age-matched controls. *P* value determined by conditional logistic regression.

There was no association between Lewis phenotype and RV1 vaccine failure (Table 3).

There was no change in observed associations when secretor phenotype was stratified by Lewis phenotype (Supplementary Table 11). In a subanalysis of secretor infants, there was no association between ABO phenotype and RV1 vaccine failure (Supplementary Tables 12 and 13).

#### HBGA Phenotype and Genotype-specific Susceptibility to RVGE

Rotavirus G or P type was confirmed in 116/119 RVGE cases. Median viral load in genotyped rotavirus cases was 1.4 × 10^7^ (IQR, 1.5 × 10^6^–4.8 × 10^7^) copies/mL. P type was confirmed in 114/119 RVGE cases.

**Figure 1.**
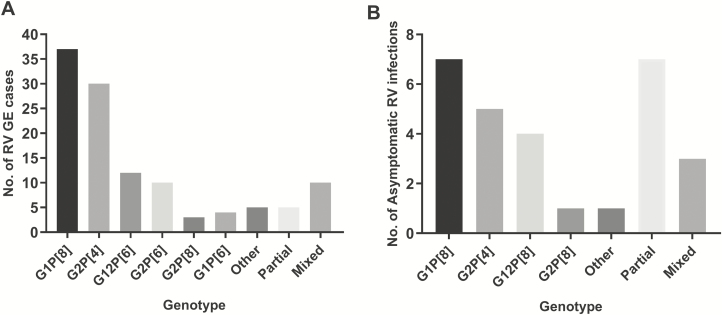
Common genotypes in rotavirus gastroenteritis (RVGE) cases and asymptomatic infection. *A*, Common genotypes in RVGE cases. *B*, Common genotypes in asymptomatic RV infection. Partial genotypes: P or G type only confirmed. Mixed infection: more than 1 G or P type identified. Abbreviation: RV, rotavirus; RVGE, rotavirus gastroenteritis.

Genotype distribution of RVGE cases is shown in Figure 1A. The 4 most common genotypes accounted for more than 75% of genotyped RVGE cases: G1P[8] (32%), G2P[4] (26%), G12P[6] (10%), and G2P[6](9%).

The prevalence of nonsecretor phenotype was significantly lower in infants with P[8] RVGE (2/47, 4%) and P[4] RVGE (2/38, 5%) compared to community controls (33/119, 28%; Table 4). All 44 infants with G1P[8] gastroenteritis were secretors. The prevalence of nonsecretor phenotype between infants with P[6] RVGE and community controls did not differ (Table 4).

**Table 4.** Histo–blood Group Antigen Phenotype Distribution in Genotype-specific Rotavirus Gastroenteritis

Histo–blood Group Antigen Phenotype	Community Controls n, %	P[8] RVGE n, % OR^a^ (95% CI) *P* Value	P[4] RVGE n, % OR (95% CI) *P* Value	P[6] RVGE n, % OR (95% CI) *P* Value
Nonsecretor	33/119, 28	2/47, 4 0.12 (0.03–0.50) .004	2/38, 5 0.17 (0.04–0.75) .02	7/33, 21 1.1 (0.42–2.7) .90
Lewis negative	31/119, 26	4/47, 9 0.26 (0.09–0.80) .02	2/38, 5 0.17 (0.04–0.73) .02	13/33, 39 3.2 (1.4–7.2) .006

Abbreviation: CI, confidence interval; OR, odds ratio; RVGE, rotavirus gastroenteritis.

^a^Odds ratio of nonsecretor/Lewis-negative phenotype in genotype-specific RVGE cases compared to community controls. *P* value determined by logistic regression.

Similarly, the prevalence of Lewis-negative phenotype was lower in infants with P[8] RVGE (4/47, 9%) and P[4] RVGE (2/38, 5%) than in community controls (31/119, 26%; Table 4). In contrast, the prevalence of Lewis-negative phenotype was higher in infants with P[6] RVGE (13/33, 39%) than in community controls (Table 4). The odds of infants being Lewis negative were increased more than 3-fold in those with P[6] RVGE (Table 4) compared to community controls.

#### HBGA Phenotype and Asymptomatic Rotavirus Infection

Asymptomatic rotavirus infection was common; 52/119 (54%) of community controls had detectable rotavirus above 100 copies/mL, with a median viral load of 628 (IQR, 258–2008) copies/mL. Due to low viral load, full genotype was only available in 21 asymptomatic infections and partial genotype in an additional 7 (Figure 1B).

The distribution of HBGA phenotypes in genotype- specific asymptomatic infection was similar to those in the wider community control population; 5/16 (31%) infants with P[8] asymptomatic infection and 3/11 (27%) infants with P[4] asymptomatic infection were nonsecretors. Three of 8 (38%) infants with G1P[8] asymptomatic infection were nonsecretors.

## DISCUSSION

Contrary to our initial hypothesis, nonsecretor phenotype was significantly less prevalent in infants with clinical vaccine failure. We found limited evidence that nonsecretor phenotype was associated with reduced vaccine take. The proportion of infants with RV1 vaccine virus shedding in the first dose period was lower in nonsecretors compared to secretors, with lower quantitative shedding on some sample days. However, the overall risk of vaccine virus shedding and peak shedding level did not differ. The proportion of infants with post-immunization RV-specific IgA seroconversion was lower in nonsecretors compared to secretors but not significantly so. Nonsecretor phenotype was associated with protection against both P[8] and P[4] RVGE, the 2 most common rotavirus strains in Malawi. Similarly, against our initial hypothesis, there was no observed association between Lewis-negative phenotype and either rotavirus vaccine take or clinical vaccine failure. Lewis-negative phenotype was less common in infants with P[8] and P[4] gastroenteritis but more common in infants with P[6] gastroenteritis, the third most common strain in this study population. These opposing effects may have brought the association between Lewis phenotype and rotavirus vaccine failure toward the null.

The lower point estimate of seroconversion in nonsecretor infants (13% compared to 27% in secretor infants) is consistent with previous studies. Bucardo et al [6] in Nicaragua reported similar findings, while Kazi et al [8] in Pakistan reported lower seropositivity following 3 doses of RV1 in nonsecretors. Our finding that nonsecretor infants are relatively protected from RVGE is consistent with data from Bangladesh where nonsecretor phenotype was associated with a decreased risk of rotavirus diarrhea in unvaccinated infants [5]. This study did not demonstrate a significant association between nonsecretor phenotype and risk of rotavirus vaccine failure, but numbers of vaccine failures were small. Our findings are also consistent with surveillance data from the United States where nonsecretors were at greatly reduced risk of vaccine failure [19], although notably in this population, 91% of gastroenteritis cases were due to P[8] infection.

Nonsecretor phenotype distribution was similar in infants with asymptomatic rotavirus infection compared to the general study population. This could suggest that nonsecretor phenotype provides relative protection against rotavirus disease, but not against asymptomatic infection. This “partial resistance” might explain the limited effect of nonsecretor phenotype on vaccine virus shedding. Asymptomatic infection could potentially allow further boosting of protective immunity [32]. We are the first to report on the relationship between HBGA phenotype and asymptomatic rotavirus infection. Although the number of infants with asymptomatic infection was high, as observed in other low-income settings [26, 33, 34], the number of genotyped asymptomatic infections was small, and conclusions should be considered within this context. However, our findings are consistent with data from Lee et al (2018) in Bangladesh, in a prospective cohort including mild diarrhea, where P[8] infection was not associated with secretor phenotype [5]. Most prior studies on the relationship between HBGA phenotype and rotavirus have focused on hospitalized RVGE. Additional data on mild and asymptomatic infections are required to confirm this partial resistance hypothesis.

Our study has several limitations. The lower than expected seroconversion rate may have limited analytic power. Exposure to wild-type rotavirus may have increased post-immunization seropositivity. However, since nonsecretors are protected against wild-type infection, any bias would be toward reduced post-immunization RV-specific IgA in this group. Subtle differences in vaccine virus shedding may have been underestimated by semiquantitative measures (Ct value), and borderline results might be clearer in a larger population. Our study relied primarily on salivary HBGA phenotyping by ELISA, which may be less sensitive than genotyping, although concordance between genotyping and phenotyping was high. Furthermore, sensitivity analysis using *FUT2* genotyping strengthened the observed protective association between nonsecretor type and odds of clinical vaccine failure.

In summary, we found little evidence in this population that nonsecretor phenotype was significantly associated with reduced vaccine take. Any possible phenotypic disadvantage in vaccine response was clearly outweighed by nonsecretors’ relative resistance to wild-type P[8] and P[4] infections, even in this population in which P[6] RVGE was common (>20%). A similar balance would likely exist in other countries with a similar or lower proportion of P[6] RVGE. Recent data show that other sub-Saharan African countries have a prevalence of P[6] RVGE that is similar to the prevalence in Malawi, while the prevalence in all other world regions is substantially lower [35, 36]. While the prevalence of P[6] could vary over time, we contend that HBGA phenotype is highly unlikely to contribute to current population differences in rotavirus vaccine effectiveness between high- and low-income countries.

## Supplementary Data

Supplementary materials are available at *Clinical Infectious Diseases* online. Consisting of data provided by the authors to benefit the reader, the posted materials are not copyedited and are the sole responsibility of the authors, so questions or comments should be addressed to the corresponding author.

ciy1067_suppl_Supplementary_Material

## Notes

***Acknowledgments.* **The authors thank the infants and their families who participated. The study was made possible by the excellent RotaRITE study team. The authors are also grateful for the support of the Malawi Ministry of Health and all clinical staff at the recruitment sites. The authors thank Professor Gagandeep Kang and her team at Christian Medical College, Vellore, India, for their generous assistance with rotavirus serology. M. I. G. is affiliated with the National Institute for Health Research (NIHR) Health Protection Research Unit in Gastrointestinal Infections at University of Liverpool in partnership with Public Health England, in collaboration with University of East Anglia, University of Oxford and the Quadram Institute. M. I. G. is based at The University of Liverpool.

***Disclaimer.* **The views expressed are those of the author(s) and not necessarily those of the National Health Service, the NIHR, the Department of Health, or Public Health England.

***Financial support.* **This work was supported by a Wellcome Trust Clinical PhD Fellowship [grant number 102464/Z/13/A to L.P.], a Wellcome Trust Programme Grant [grant number 091909/Z/10/Z] and the MLW Programme Core Grant Strategic Award [grant number 101113/Z/13/Z].

***Potential conflicts of interest.* **K. C. J. and N. B.-Z. have received research grant funding from GlaxoSmithKline (GSK). N. B.-Z. also received funding from Takeda Pharmaceuticals. M. I. G. receives research grant funding from GSK, Merck, Takeda, and Sanofi-Pasteur and Merck, Sharpe and Dohme Corporation (SPMSD). N. A. C. has received research grant funding and honoraria for participation in independent data monitoring committees from GSK. All other authors: No reported conflicts. All authors have submitted the ICMJE Form for Disclosure of Potential Conflicts of Interest. Conflicts that the editors consider relevant to the content of the manuscript have been disclosed.
