# Supplementary material for: Nonsecretor Histo–blood Group Antigen Phenotype Is Associated With Reduced Risk of Clinical Rotavirus Vaccine Failure in Malawian Infants
Source: Clin Infect Dis. 2018 Dec 18;69(8):1313–9. doi: 10.1093/cid/ciy1067 (PMC6763638; doi:10.1093/cid/ciy1067)
Supplement: ciy1067_suppl_Supplementary_Material [file ciy1067_suppl_supplementary_material.docx]

**Supplementary Tables**

Supplementary Table 1: Demographic characteristics of cohort participants

| Characteristic | Shedding  cohort n=202 | Seroconversion cohort n=196 | Defaulted  n=60 |
| --- | --- | --- | --- |
| Infant characteristics |  |  |  |
| Male | 101 (50%) | 94 (48%) | 29 (48%) |
| Exclusively breastfed | 201 (99%) | 195 (99%) | 60 (100%) |
| HIV-exposed | 28/201(14%) | 29/195 (15%) | 6 (10%) *(n=59)* |
| Low birth weight (<2.5kg) | 21/197(11%) | 22/190 (12%) | 4 (7%) *(n=58)* |
| Nutritional status |  |  |  |
| Median weight for age  z-score at 1^st^ RV1 dose (IQR) | -0.38(-1.04-0.26) | -0.46 (-1.2-0.31) | -0.34 (-1.04-0.39) |
| Median length for age z-score at 1^st^ RV1 dose (IQR) | -0.69(-1.83- **-**0.03) (n=199) | -0.70 (-1.88- **-**0.02) n=190 | -0.30 (-1.30-0.42) |
| Median weight for length z-score at 1^st^ RV1 dose | 0.5(-0.45-1.33) | 0.39 (-0.52-1.35) n=190 | 0.18 (-0.60-1.2) |
| Sanitation and socioeconomic predictors | | | |
| Median household size (IQR) | 5 (4-6) | 5 (4-6) | 4 (3-5) |
| Non-piped water source | 22 (11%) | 23 (12%) | 9 (15%) |
| Pit-latrine type toilet | 180/199 (91%) | 176/194 (91%) | 52/57 (91%) |
| Electricity at home | 105 (52%) | 98 (50%) | 35 (58%) |
| One or more household member with salary | 163 (81%) | 155 (79%) | 46 (77%) |
| Household food insecurity | 70 (30%) | 64 (33%) | 16 (27%) |
| Median years of maternal education (IQR) | 10 (8-12) | 10 (8-12) | 10 (8-12) |

*233 infants completed follow-up. Of these infants, 202 infants had complete shedding data, 196 infants had complete seroconversion data, 165 infants had complete data for both shedding and seroconversion. 60 infants did not complete follow-up (defaulted or withdrew).*

Supplementary Table 2: Comparison of demographic characteristics between infants with complete and incomplete data

| Characteristic | Complete data*  n=165 | Incomplete data**  n=128 | p† |
| --- | --- | --- | --- |
| Infant characteristics |  |  |  |
| Male | 78 (47%) | 68 (53%) | 0.32 |
| Exclusively breastfed | 164 (99%) | 128 (100%) | 0.38 |
| HIV-exposed | 25/164 (15%) | 13/127 (10%) | 0.21 |
| Low birth weight (<2.5kg) | 17/160 (11%) | 13/125 (10%) | 0.95 |
| Nutritional status |  |  |  |
| Median weight for age  z-score at 1^st^ RV1 dose (IQR) | -0.49 (-1.2-0.26) | -0.27 (-0.95-0.36) | 0.16 |
| Median length for age z-score at 1^st^ RV1 dose (IQR) | -0.83 (-1.99- **-**0.03) (n=162) | -0.40 (-1.26-0.39)  (n=125) | <0.01 |
| Median weight for length z-score at 1^st^ RV1 dose | 0.49 (-0.41-1.35) n=162 | 0.17 (-0.72-1.2) | 0.14 |
| Median household size (IQR) | 5 (4-6) | 4 (4-6) | 0.38 |
| Non-piped water source | 18 (11%) | 18 (14%) | 0.42 |
| Pit-latrine type toilet | 149/164 (91%) | 110/122 (90%) | 0.84 |
| Electricity at home | 83 (50%) | 72 (56%) | 0.31 |
| One or more household member with salary | 132 (80%) | 100 (78%) | 0.70 |
| Household food insecurity | 56 (34%) | 30 (23%) | 0.05 |
| Median years of maternal education (IQR) | 10 (8-12) | 10 (8-12) | 0.08 |

**Complete data= complete data for both shedding and seroconversion. **Incomplete data, incomplete data for either seroconversion or shedding (68 infants) or defaulted/withdrew (60 infants). †p values refer to Wilcoxon rank sum test for comparison of median values and Chi squared test for comparison of proportions.*

Supplementary Table 3: Vaccine virus shedding by sample day and HBGA phenotype

|  | **n^a^, % vaccine virus shedding** | | | | | |
| --- | --- | --- | --- | --- | --- | --- |
|  | **Non-secretor** | **Secretor** | **RR (95%CI)**  **p value^b^** | **Lewis negative** | **Lewis positive** | **RR (95%CI)**  **p value^b^** |
| **1^st^ dose period** | | | | | | |
| **Day 4** | 10/38,  21 | 53/159, 33 | 0.63 (0.35-1.1)  0.12 | 14/40,  35 | 49/167,  29 | 1.2 (0.74-1.9)  0.48 |
| **Day 6** | 12/48,  25 | 58/172, 34 | 0.74 (0.44-1.3) 0.27 | 16/46,  35 | 54/174, 31 | 1.1 (0.71-1.8)  0.62 |
| **Day 8** | 12/50,  24 | 51/164, 31 | 0.77 (0.45-1.3)  0.35 | 15/48,  31 | 48/166, 29 | 1.1 (0.67-1.75)  0.75 |
| **Day 10** | 4/49,  8 | 51/182, 28 | 0.29 (0.11-0.77)  0.01 | 9/41,  22 | 46/190, 24 | 0.91 (0.48-1.7)  0.76 |
| **2^nd^ dose period** | | | | | | |
| **Day 4** | 16/40,  40 | 50/147, 34 | 1.2 (0.76-1.8) 0.48 | 13/38,  34 | 53/149, 36 | 0.96 (0.59-1.6)  0.88 |
| **Day 6** | 10/31,  24 | 51/153, 33 | 0.73 (0.41-1.3) 0.29 | 13/42,  31 | 48/152, 32 | 0.98 (0.59-1.6)  0.94 |
| **Day 8** | 9/34,  21 | 41/106, 28 | 0.75 (0.40-1.4) 0.38 | 10/41,  24 | 40/149, 27 | 0.91 (0.50-1.7)  0.75 |
| **Day 10** | 7/42,  17 | 36/150,  24 | 0.69 (0.33-1.4) 0.33 | 4/39,  10 | 39/153, 25 | 0.40 (0.15-1.1)  0.07 |

*a. A total of 243 infants provided at least two samples in the 1^st^ dose period, and 214 infants in the 2^nd^ dose period. Not all infants provided samples at each time point, therefore numbers of samples vary by sample day. b. Determined by binomial logistic regression.*

Supplementary Table 4: Vaccine virus shedding Ct value by sample day and HBGA phenotype

|  | **Ct value, median (IQR)** | | | | | |
| --- | --- | --- | --- | --- | --- | --- |
|  | **Non-secretor** | **Secretor** | **p^a^** | **Lewis negative** | **Lewis positive** | **p^a^** |
| **1^st^ dose period** | | | | | | |
| **Day 4** | 34.7 (28.3-37.8) | 35.1 (30.9-37.1) | 0.61 | 33.0 (29.1-36.5) | 35.2 (31.5-37.2) | 0.39 |
| **Day 6** | 34.9 (33.2-37.6) | 31.8 (29.2-35.7) | 0.02 | 33.2 (30.5-35.8) | 32.3 (29.2-35.7) | 0.43 |
| **Day 8** | 34.4 (31.4-37.6) | 30.0 (25.9-34.2) | 0.02 | 33.0 (29.0-37.3) | 30.4 (26.1-34.0) | 0.16 |
| **Day 10** | 32.6 (27.7-35.7) | 32.2 (29.2-35.1) | 0.94 | 34.7 (29.9-34.9) | 32.2 (29.2-35.1) | 0.51 |
| **2^nd^ dose period** | | | | | | |
| **Day 4** | 35.1 (33.8-37.3) | 34.4 (32.3-37.3) | 0.26 | 36.6 (34.3-38.0) | 34.6 (32.3-37.1) | 0.08 |
| **Day 6** | 34.1 (33.1-37.1) | 34.6 (32.0-35.8) | 0.94 | 35.5 (33.3-35.9) | 34.3 (32.0-35.8) | 0.55 |
| **Day 8** | 35.0 (34.6-36.1) | 33.8 (30.7-36.6) | 0.12 | 34.5 (33.5-37.0) | 33.9 (30.8-36.4) | 0.36 |
| **Day 10** | 37.2 (32.5-37.6) | 34.4 (32.3-35.3) | 0.22 | 35.2 (33.2-37.0) | 34.4 (32.1-35.4) | 0.53 |

*a. Determined by Wilcoxon rank-sum test.*

Supplementary Table 5: Cohort sensitivity analysis with confirmatory FUT2 genotyping

|  | Secretor | Non-secretor* | p value† |
| --- | --- | --- | --- |
| Vaccine virus shedding  n, %, RR | 90/167, 54 (46-61%)  1.4 (0.9-2.1) | 14/35, 40 (25-58%)  0.7 (0.5-1.1) | 0.14 |
| Seroconversion  n, %, RR | 42/160, 26 (20-34%)  1.9 (0.8-4.4) | 5/36, 14 (6-30%)  0.5 (0.2-1.2) | 0.12 |

** Fifty infants included in shedding or seroconversion cohorts were phenotypic non-secretors at 6 weeks. Of these infants, 3 had heterozygous secretor genotype at 10 weeks, a further 7 infants were phenotypic secretors at 7 weeks. For this sensitivity analysis, these infants were recategorized as secretors. Of the remaining 40 infants, 12 had no blood available for FUT2 genotyping but were confirmed as phenotypic non-secretors at 10 weeks. 28 were confirmed as non-secretors on FUT2 genotyping. For this sensitivity analysis, these infants remained categorised as non-secretors. †Chi squared test.*

Supplementary Table 6: Vaccine virus shedding and seroconversion by stratified HBGA phenotype

| HBGA Phenotype | Vaccine virus shedding  n, % (95%CI) | RR  (95%CI)  p value | Seroconversion  n, % (95%CI) | RR  (95%CI)  p value |
| --- | --- | --- | --- | --- |
| Non-secretor |  |  |  |  |
| *Lewis positive* | 10/30  33 (18-53%) | 0.63 (0.31-1.2)  p=0.18 | 4/29  14 (5-33%) | 1.1 (0.23-5.4)  p=0.90 |
| *Lewis negative* | 8/15  53 (27-78%) |  | 2/16  13 (3-43%) |  |
| Secretor |  |  |  |  |
| *Lewis positive* | 74/129  57 (49-66%) | 1.4 (0.85-2.1)  p=0.21 | 31/120  26 (19-35%) | 0.80 (0.44-1.4)  p=0.46 |
| *Lewis negative* | 12/28  43 (25-62%) |  | 10/31  32 (18-51%) |  |

*n=number, %=percent, RR=risk ratio of vaccine virus faecal shedding/seroconversion in Lewis positive non-secretor compared to Lewis negative non-secretor infants, and in Lewis positive secretor compared to Lewis negative secretor infants.*

Supplementary Table 7: Vaccine virus shedding and seroconversion by ABO phenotype (secretor infants only)

| HBGA Phenotype^a^ | Vaccine virus shedding  n, % (95%CI) | RR^b^  (95%CI)  p value | Seroconversion  n, % (95%CI) | RR  (95%CI)  p value |
| --- | --- | --- | --- | --- |
| O | 35/67  52 (40-64%) | ref | 14/65  22 (13-34%) | ref |
| A | 28/45  62 (47-76%) | 1.2 (0.86-1.6)  p=0.29 | 16/49  33 (21-47%) | 1.5 (0.82-2.8)  p=0.18 |
| B | 20/37  54 (37-70%) | 1.0 (0.71-1.5)  p=0.86 | 9/30  30 (16-49%) | 1.4 (0.68-2.9)  p=0.37 |
| AB | 3/8  38 (9-79%) | 0.72 (0.29-1.8)  p=0.48 | 2/7  29 (4-78%) | 1.3 (0.38-4.7)  p=0.66 |

*a. Secretor infants only n=157 for vaccine virus shedding analysis, n=151 for seroconversion analysis. ABO type cannot be determined from saliva in non-secretors. b. Risk ratio of vaccine virus shedding/seroconversion in type A/B/AB secretor infants compared to type O secretor infants*

Supplementary Table 8: Vaccine virus shedding and seroconversion by ABO phenotype: Type O compared to non-O.

| HBGA Phenotype^a^ | Vaccine virus shedding  n, % (95%CI) | RR^b^  (95%CI)  p value | Seroconversion  n, % (95%CI) | RR  (95%CI)  p value |
| --- | --- | --- | --- | --- |
| O | 35/67  52 (40-64%) | 0.92 (0.69-1.2)  p=0.59 | 14/65  22 (13-34%) | 0.69 (0.39-1.2)  p=0.19 |
| Non-O | 51/90  57 (46-67%) |  | 27/86  31 (22-42%) |  |

*a. Secretor infants only n=157 for vaccine virus shedding analysis, n=151 for seroconversion analysis. ABO type cannot be determined from saliva in non-secretors. b. Risk ratio of vaccine virus shedding/seroconversion in type O secretor infants compared to non-O type (A, B, or AB) secretor infants*

Supplementary Table 9: Characteristics of RV GE cases and community controls

| Characteristic | RV GE  cases  n=119 | | | Community Controls  n=119 | | | | p^†^ |
| --- | --- | --- | --- | --- | --- | --- | --- | --- |
| Infant Characteristics | |  | | | |  |  |  |
| Mean age in months at recruitment, (95%CI) | 9.0 (8.6-9.3) | | | 9.7 (9.3-10.1) | | | | 0.01 |
| Male | 72, 61% | | | 61, 51% | | | | 0.15 |
| HIV-exposed^*^ | 17, 14% | | | 19, 16% | | | | 0.72 |
| Low birth weight (<2.5kg) | 14/111, 13% | | | 11/117, 9% | | | | 0.44 |
| Nutritional Status |  | | |  | | | |  |
| Median weight for age  z-score (IQR)^**^ | -0.38 (-1.40-0.47) | | | -0.43(-1.04-0.39) | | | | 0.85 |
| Median length for age  z-score (IQR) | -0.68(-1.77-0.93)  *n=95* | | | -0.74 (-1.99-0.10)  *n=95* | | | | 0.25 |
| Median weight for length z-score (IQR)^**^ | -0.60(-1.59-0.38)  *n=95* | | | -0.05(-1.38-0.87)  *n=95* | | | | 0.14 |
| Median MUAC, cm (IQR) | 13.1 (12.4-14.0) | | | 13.8 (13.2-14.5) | | | | <0.001 |
| Sanitation and socioeconomic factors | | |  | |  |  |  |  |
| Median household size (IQR) | 5 (4-6) | | | 4 (3-6) | | | | 0.12 |
| Non-piped water source | 22, 19% | | | 17, 14% | | | | 0.37 |
| Pit-latrine type toilet | 115, 97% | | | 114, 96% | | | | 0.73 |
| Electricity at home | 60, 50% | | | 54, 45% | | | | 0.44 |
| One or more household members with salary | 82, 69% | | | 75, 63% | | | | 0.34 |
| Median years of maternal education (IQR) | 8 (5-11) | | | 9 (7-11) | | | | 0.25 |

*Denominator n=119 for both cases and controls unless stated otherwise. †p values refer to t-test for comparison of means, Wilcoxon rank sum test for comparison of median values and Chi squared test for comparison of proportions. *11/17 HIV exposed RV GE cases and 15/19 HIV exposed community controls had a negative HIV DNA PCR at 6 weeks old. One community control was known HIV infected and on ART. Status of remaining HIV exposed infants was unknown. **Weight adjusted for dehydration status by adding 5% for some dehydration and 10% for severe dehydration. Length data not available for all infants.*

Supplementary Table 10: Sensitivity analysis of case-control study by FUT2 genotyping

| HBGA phenotype | Prevalence in  RV GE cases (Vaccine failures)  n, % (95%CI) | Prevalence in Community Controls  n, % (95%CI) | Odds ratio^b^ (95%CI)  p value |
| --- | --- | --- | --- |
| Non-secretor^a^ | 11/119  9 (5-16%) | 29/119  24 (17-33%) | 0.36 (0.17-0.74) p=0.002 |

*a. FUT2 genotype was determined for 38/47 phenotypic non-secretor infants included in the primary analysis: 7/38 (19%) of phenotypic non-secretor infants tested (3 rotavirus gastroenteritis cases, 4 community controls) had a secretor genotype (2 homozygous and 5 heterozygous). For this sensitivity analysis, these 7 infants were recategorized as secretors. 9 non-secretor infants had no blood available for FUT2 genotyping. These infants remained categorized as non-secretors. For this re-analysis the total number of infants categorized as non-secretor was therefore 40. b. Odds ratio of non-secretor genotype in vaccine failures compared to age-matched controls, p value determined by conditional logistic regression.*

Supplementary Table 11: Stratified HBGA phenotype distribution in rotavirus vaccine failures and community controls

| HBGA Phenotype | Prevalence in RVGE cases (Vaccine Failures)  n, % (95%CI) | Prevalence in Community Controls  n, % (95%CI) | Odds ratio^a^  (95%CI)  p value |
| --- | --- | --- | --- |
| Non-secretor |  |  |  |
| *Lewis positive* | 6/14  43 (18-72%) | 22/33  67 (48-81%) | 0.34 (0.10-1.4)  p=0.134 |
| *Lewis negative* | 8/14  57 (28-82%) | 11/33  33 (19-52%) |  |
| Secretor |  |  |  |
| *Lewis positive* | 89/105  85 (76-91%) | 66/86  77 (66-84%) | 1.7 (0.81-3.5)  p=0.16 |
| *Lewis negative* | 16/105  15 (9-24%) | 20/86  23 (15-34%) |  |

*n=number, %=percent, a. Odds ratio of Lewis positive non-secretor phenotype compared to Lewis negative non-secretor phenotype in vaccine failures compared to community controls and odds ratio of Lewis positive secretor phenotype compared to Lewis negative secretor phenotype in vaccine failures compared to community controls. Determined by logistic regression (unmatched analysis) as insufficient discordant pairs for conditional logistic regression.*

Supplementary Table 12: ABO phenotype distribution in rotavirus vaccine failures and community controls (secretor infants only)

| HBGA Phenotype^a^ | Prevalence in RVGE cases (vaccine failures)  n, % (95%CI) | Prevalence in community controls  n, % (95%CI) | Odds ratio^b^  (95%CI)  p value |
| --- | --- | --- | --- |
| O | 47/105  45 (35-55%) | 34/86  40 (30-50%) | ref |
| A | 29/105  28 (20-37%) | 22/86  26% (17-36) | 0.80 (0.38-1.7)  p=0.55 |
| B | 26/105  25 (17-34%) | 25/86  29 (20-40%) | 0.57 (0.24-1.35)  p=0.20 |
| AB | 3/105  3 (0.9-9%) | 5/86  6 (2-13%) | 0.17 (0.01-1.7)  p=0.13 |

*n= number, % percent. a. Secretor infants only. n=105 vaccine failures n=86 community controls. b. Odds ratio of type O phenotype compared to type A/B/AB phenotype in secretor infants with vaccine failure compared to secretor community controls. Determined by conditional logistic regression.*

Supplementary Table 13: Type O versus non-O phenotype distribution in rotavirus vaccine failures and community controls (secretor infants only)

| HBGA Phenotype^a^ | Prevalence in RVGE cases (vaccine failures)  n, % (95%CI) | Prevalence in community controls  n, % (95%CI) | Odds ratio^b^  (95%CI)  p value |
| --- | --- | --- | --- |
| O | 47/105  45 (35-55%) | 34/86  40 (30-50%) | 1.5 (0.77-2.8)  p=0.25 |
| Non-O | 58/105  55 (45-65%) | 52/86  60% (50-70%) |  |

*n= number, % percent. a. Secretor infants only. n=105 vaccine failures n=86 community controls. b. Odds ratio of type O phenotype compared to non-O (type A/B/AB phenotype) in secretor infants with vaccine failure compared to secretor community controls. Determined by conditional logistic regression.*

**Supplementary Methods**

**Sample Size Calculation**

Sample size for the cohort study was based on the assumptions that population prevalence of non-secretor/Lewis negative phenotype would be 20%, that the proportion of infants with seroconversion would be 50% and that the proportion of infants with vaccine virus shedding would be 30%. A sample size of 200 was required to achieve 80% power to detect a risk ratio of 0.5 (versus equal risk, alpha 0.05).

Sample size for the case-control study was based on the estimated proportion of non-secretor/Lewis negative phenotype in controls, assumed to be 20% [32]. With 1:1 controls, a sample size of 123 cases was required to achieve 80% power to detect an odds ratio of 2.5 (versus equal odds, alpha 0.05).

**VP6 and NSP2 PCR**

Primers and probes used in VP6 qRT-PCR and NSP2 RT-PCR are detailed in Supplementary Methods Table 1. Reaction mix was prepared with 12.5μl PCR Mastermix (Low Rox), 0.5μl of each primer (at 20pmol/ μl), 0.25μl probe (20μM), 8.75μl nuclease free water. Reaction mix (22.5 μl) and cDNA(2.5μl) were added to a 96 well FAST plate. Cycling conditions were: 95°C for 2 minutes, then 40 cycles of 95°C for 15 seconds, 60°C for 1 minute.

Supplementary Methods Table 1: Probes and primers for VP6 qRT-PCR and NSP2 RT-PCR

| Primer/Probe | Sequence (5’-3’) | Nucleotide Positions |
| --- | --- | --- |
| VP6F | GAC GGV GCR ACT ACA TGG T | 747-766 |
| VP6R | GTC CAA TTC ATN CCT GGT G | 1126-1106 |
| VP6Probe | ^FAM^ CCA CCR AAY ATG ACR CCA GCN GTA^MGB^ | 912-935 |
| RV1NSP2-F | GAACTTCCTT GAATATAAGA TCACACTGA | 546-574 |
| RV1NSP2-R | TTGAAGACGT AAATGCATAC CAATTC | 826-801 |
| RV1NSP2-Probe | ^FAM^-TCCAATAGAT TGAAGTCAGTAACGTTTCCA-^BHQ1^ | 782-753 |

***ABO and Lewis Phenotyping ELISA***

Saliva samples were diluted 1:1000 in 0.05M Carbonate Bicarbonate coating buffer (pH 9.6 ±0.2). 100µl of diluted saliva was added to each well of a 96 well plate (Nunc-Microsorp) and incubated overnight at 4°C. For blanks and negative controls, 100µl of coating buffer was added. The following morning, test plates were incubated for 2 hours at 37°C in a moist chamber. Plates were washed in PBS-T then 1% skim milk blocking buffer (200µl) was added to each well and plates incubated for 1h at 37°C. Monoclonal antibodies were diluted in 0.2% skim milk dilution buffer. Anti-A (HE-103, Thermo-Fisher Scientific), Anti-B (89-F, Thermo-Fisher Scientific) and Anti-H (97-I, Thermo-Fisher Scientific) antibodies were diluted 1:2000. Anti-Leb (Seraclone 808423, Bio-Rad) were diluted 1:500. Anti-Lea antibodies (Seraclone 808404, Bio-Rad) were diluted 1: 1000. Diluted primary antibodies (100µl) were added to each well of the appropriate test plate and incubated for 1.5 hours at 37°C in a moist chamber. Enzyme-conjugated secondary antibody Anti-Mouse IgM – peroxidase antibody produced in goat (SIGMA-ALDRICH) was diluted 1:5000 in dilution buffer. 100µl of diluted secondary antibody was added to each well and incubated for 1.5 hours at 37°C in a moist chamber. Finally, enzyme substrate 3’,3’,5’,5’-tetramethylbenzidine, supersensitive, for ELISA (SIGMA-ALDRICH) (100µl) was added to each well and incubated for 10 minutes in darkness. The reaction was stopped by 100µl of 1M H_2_SO_4_ to each well and absorbance read at 450nm using a Biotek microtitre plate reader. The cut-off OD for each HBGA ELISA was 3x the average value of the blanks and negative controls.

***Lectin phenotyping ELISA***

Saliva samples were diluted 1:1000 in 0.05M Carbonate Bicarbonate coating buffer (pH 9.6 ±0.2). 100µl of diluted saliva was added to each well of a 96 well plate (Nunc-Microsorp). Test plates were incubated for 2 hours at 37°C then overnight at 4°C. The remaining steps were the same as described above. Lectin conjugated antibodies (L8146, Sigma-Aldrich) were diluted 1:3200 in 0.02% skim milk dilution buffer.

***Rotavirus serology***

RV-specific IgA was determined by sandwich ELISA [26]. Rotavirus (WC3-infected MA104 cell culture lysates) was bound with rabbit anti-rotavirus IgG (provided by Christian Medical College, Vellore, India). Uninfected MA104 cell lysates were included for background correction. Rotavirus-specific IgA was detected using biotin-conjugated rabbit anti-human IgA, with avidin-biotin-peroxidase complex and peroxidase substrate. Quantification was made by comparison to a standard plasma [27] and reported as geometric mean concentration (GMC) in units per litre (U/mL).

***HBGA FUT2 Genotyping: PCR and gel electrophoresis***

The FUT2 gene expression determines secretor phenotype. Inactivating mutations of FUT2 result in a non-secretor phenotype. Amplification of the FUT2 gene was performed by PCR (primers detailed in Supplementary Methods Table 2), and restriction fragment length polymorphism used to identify inactivating mutations. The most common mutation, “G428A” affects restriction sites for the enzyme AvaII (G’GWCC where W=A or T). AvaII was used to cut the PCR product, and fragments examined using gel electrophoresis. Homozygous secretors (two functional copies of FUT2 gene) were identified by one 136 base pair and one 59 base pair fragment. Homozygous non-secretors (two copies of FUT2 gene with inactivating mutation) were identified by unrestricted PCR product (195 base pairs). Heterozygous secretors (one functional copy, one inactivating mutation) were identified by three bands: unrestricted PCR product (195 base pairs), and two fragments (136 and 59 base pairs).

Supplementary Methods Table 2: Primers for FUT2 PCR

| Primer/Probe | Sequence (5’-3’) |
| --- | --- |
| FUT2 F | GAGGAATACCGCCACATCCCGGGGGAGTAC |
| FUT2 R | ATGGACCCCTACAAAGGTGCCCGGCCGGCT |

FUT2 PCR mastermix was prepared with 10X buffer (5 µl), 50mM Magnesium sulphate (2.5 µl), dNTPs (1 µl), Taq polymerase 5U/µl (0.3µl), each primer (1.0 µl) and nuclease free water (34.2 µl). Mastermix (45µl) and 5µl of extracted DNA was then added to each PCR tube. Cycling conditions were 95°C for 5minutes, then 30 cycles of 94°C for 1 minute, 45°C for 2 minutes, 72°C for 1 minute. Finally, 72°C for 7 minutes then hold at 15°C.

Mastermix for the restriction step was prepared with 10X CutSmart (3 µl), AvaII (1µl) and nuclease free water (21µl) per reaction. Mastermix (25µl) and 5µl of extracted DNA was then added to each PCR tube. Tubes were briefly centrifuged and incubated at 37°C for 15 minutes then inactivated at 80°C for 20 minutes. Agarose gel electrophoresis was then used for visualization and identification of DNA fragments.
